# matUtils: Tools to Interpret and Manipulate Mutation Annotated Trees

**DOI:** 10.1101/2021.04.03.438321

**Published:** 2021-04-04

**Authors:** Jakob McBroome, Bryan Thornlow, Angie S. Hinrichs, Nicola De Maio, Nick Goldman, David Haussler, Russell Corbett-Detig, Yatish Turakhia

**Affiliations:** 1. Department of Biomolecular Engineering, University of California Santa Cruz. Santa Cruz, CA 95064, USA; 2. Genomics Institute, University of California Santa Cruz, Santa Cruz, CA 95064, USA; 3. European Molecular Biology Laboratory, European Bioinformatics Institute (EMBL-EBI), Wellcome Genome Campus, Cambridge CB10 1SD, UK

## Abstract

**Motivation:**

Standard phylogenetics workflows have struggled to meet the demands of the COVID-19 pandemic. Efficient data storage formats that enable rapid placement and accession have been developed, but additional tools are required to make them broadly accessible.

**Results:**

matUtils is a toolkit for the manipulation and analysis of mutation-annotated trees (MAT) to support pandemic-related research and data sharing.

**Availability and Implementation:**

matUtils software is available at https://github.com/yatisht/usher and our daily updated database of MAT files for public SARS-CoV-2 sequences is available at https://hgwdev.gi.ucsc.edu/~angie/UShER_SARS-CoV-2/.

## 1 Introduction

**Figure 1:**
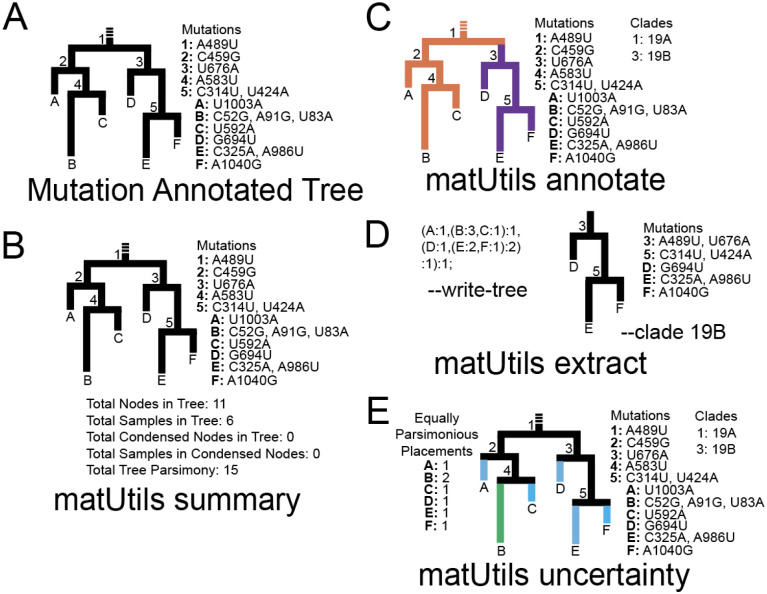
matUtils functions enable fast, user-friendly editing of mutation-annotated trees (MATs). **(A)**: An example MAT with tree topology corresponding to the MAT on the left and the mutation annotations on each node shown on the right. **(B):** *matUtils summary* outputs sample-, clade-, and tree-level statistics for the input MAT. **(C):** *matUtils annotate* allows the user to annotate internal nodes with clade names. In this example, nodes 1 and 3 are annotated with clade names 19A and 19B, respectively. **(D):** *matUtils extract* allows conversion of a MAT (of panel C in this example) to a Newick format (left) or subset the MAT for a specified clade (right), among other functions. **(E)**: *matUtils uncertainty* outputs parsimony scores and equally parsimonious placements for each sample.

Phylogenetic analyses are at the core of tracing viral evolution and interpreting local and global transmission networks but the vast scale of available SARS-CoV-2 genome sequences has revealed deep inadequacies in existing phylogenetic workflows, data sharing platforms and data formats. Specifically, typical phylogenetic frameworks use a multiple sequence alignment (MSA) in FASTA format and use the variation data it encodes to produce a phylogenetic tree, but as the number of available SARS-CoV-2 genomes approaches million-scale, sharing global SARS-CoV-2 variation data using FASTA or Variant Call Format (VCF) has become challenging. Moreover, interpretation of data using separate formats for phylogenetic trees (Newick) and variation data (VCF) is also getting more complex and cumbersome. New data formats, such as mutation-annotated tree (MAT) (Turakhia, Thornlow, *et al.*, 2020) or succinct tree sequences (Kelleher et al 2019), that combine tree and sequence data in efficient data structures can greatly alleviate these challenges. Of particular interest is the MAT, a file format which stores a phylogenetic tree in which the branches are annotated with the mutations that are inferred to have occurred on them (Figure 1A). MAT also allows rapid placement of new samples onto existing phylogenetic trees using maximum parsimony (Turakhia, Thornlow, *et al.*, 2020), but future extensions of this format would also support likelihood calculations. Here, we present a database of MATs produced during the SARS-CoV-2 pandemic and matUtils, a command line program for manipulating, querying, and converting MAT-formatted files.

## 2 Implementation

MAT is an extremely efficient data format that is capable of facilitating data sharing during the pandemic even as genome sequence datasets become extremely large (Turakhia, Thornlow, *et al.*, 2020). In a MAT, mutations are annotated only once on the ancestral branch in which they occur rather than once for each of its descendant samples, such as in VCF format. Thus, MAT provides a type of “evolutionary compression”. MATs use protocol buffers (https://developers.google.com/protocol-buffers) for serializing structured data (Turakhia, Thornlow et al, 2020). Indeed, for a tree of 400,000 SARS-CoV-2 genomes, a MAT requires just 31 MB rather than 12 GB for a MSA or 21 GB for a VCF. This makes MAT an appealing option for sharing SARS-CoV-2 phylogenetic and variation data simultaneously, especially since our most recent MAT format (as of UShER v0.2.0) also allows annotating ancestral branches with clade labels in addition to mutations. As a part of this work, we are maintaining and openly sharing an up-to-date MAT database containing global SARS-CoV-2 sequences from public databases (GenBank (https://www.ncbi.nlm.nih.gov/genbank/), COG-UK (https://www.cogconsortium.uk/) and China National Center for Bioinformation (https://bigd.big.ac.cn/ncov/)), including annotations for Nextstrain clades (Hadfield et al 2018) and Pangolin lineages (Rambaut *et al.*, 2020). These MATs can be obtained from https://hgwdev.gi.ucsc.edu/~angie/UShER_SARS-CoV-2/.

To accompany this database, we present matUtils, a toolkit for performing operations on MATs for rapid interpretation and analysis in genomic surveillance and contact tracing efforts. matUtils is implemented in C++ and included in the UShER package (Turakhia, De Maio, *et al.*, 2020), which can be installed using bioconda (Grüning *et al.*, 2018). matUtils provides speed and efficiency that scales easily to million sample phylogenies (Table S1–5).

## 3 Usage

matUtils provides four core functions: summary, annotate, extract and uncertainty (Figure 1). We provide detailed instructions for the usage of each module on our wiki (https://usher-wiki.readthedocs.io/).

### 3.1 Summary:

This function provides a brief summary of the available data in the input MAT file and is meant to serve as a typical first step in any MAT analysis. In particular, this function provides a count of the total number of samples in the MAT, the number of annotated clades, size of each clade, the total parsimony score (i.e. the sum of mutation events on all branches of the MAT), and the number of distinct mutations, among other statistics.

### 3.2 Annotate:

One of the central uses of phylogenetics during the pandemic is to trace the emergence and spread of new viral lineages. We support this by providing a method for automatically identifying clade roots based on user-provided clade assignments, such as the nextstrain clades (Hadfield et al 2018). These labels will propagate to descendent samples within the MAT, allowing clade members to be queried or manipulated by UShER and other commands described below. Our annotate method guarantees that clades remain monophyletic in a MAT and its implementation performs comparably to state-of-the-art tools with a similar functionality for assigning clades to Newick format trees (Table S1).

### 3.3 Extract:

Many SARS-CoV-2 phylogenetic analyses require focussing on a phylogeny of a specific subset of samples rather than a comprehensive global phylogeny. To aid this, the matUtils extract function accepts a set of samples or sample selection criteria (*e.g*. samples which contain a specified mutation, or have less than a specified parsimony score) and produces a smaller MAT containing only those samples. The extract function also provides an option to output a Newick, a VCF or an Auspice-compatible JSON file corresponding to the specified samples. Our implementation compares well to other tools for subsetting Newick trees (Hubisz *et al.*, 2011; Junier and Zdobnov, 2010; Danecek *et al.*, 2021) (Table S2–5) even though MAT also contains genetic variation information. Because the MAT representation of genetic variation is much more efficient than VCF and MSA, matUtils is also much faster than variation subsetting tools. For example, matUtils is an order of magnitude faster than bcftools (Li, 2011) to extract 1,500 samples containing a specific mutation from a file containing 400,000 samples (Table S2).

### 3.4 Uncertainty:

A fundamental concern in phylogenies is topological uncertainty. This is especially true for public health, where sample level uncertainty statistics can represent the reliability of contract tracing. The uncertainty function of matUtils computes the number of equally parsimonious placements and the branch parsimony score (Turakhia, De Maio, *et al.*, 2020) that exist for each specified sample in the input MAT. This is accomplished by mapping a virtual copy of the sample to the tree, disallowing mapping directly to the original sample. The output file is compatible as “drag-and-drop” metadata with the Auspice visualization platform (Hadfield *et al.*, 2018).

## 4 Conclusion

We present a new optimized toolkit for the manipulation and conversion of mutation-annotated trees (MAT), an efficient format for sharing and analyzing phylogenetic and genome variation data simultaneously. This will facilitate rapid data sharing and phylogenetic analysis during the ongoing SARS-CoV-2 pandemic and into the future.

## Supplementary Material

Supplement 1
