# A daily-updated database and tools for comprehensive SARS-CoV-2 mutation-annotated trees

**DOI:** 10.1101/2021.04.03.438321

**Published:** 2021-07-13

**Authors:** Jakob McBroome, Bryan Thornlow, Angie S. Hinrichs, Nicola De Maio, Nick Goldman, David Haussler, Russell Corbett-Detig, Yatish Turakhia

**Affiliations:** 1. Department of Biomolecular Engineering, University of California Santa Cruz. Santa Cruz, CA 95064, USA; 2. Genomics Institute, University of California Santa Cruz, Santa Cruz, CA 95064, USA; 3. European Molecular Biology Laboratory, European Bioinformatics Institute (EMBL-EBI), Wellcome Genome Campus, Cambridge CB10 1SD, UK

## Abstract

The vast scale of SARS-CoV-2 sequencing data has made it increasingly challenging to comprehensively analyze all available data using existing tools and file formats. To address this, we present a database of SARS-CoV-2 phylogenetic trees inferred with unrestricted public sequences, which we update daily to incorporate new sequences. Our database uses the recently-proposed mutation-annotated tree (MAT) format to efficiently encode the tree with branches labeled with parsimony-inferred mutations as well as Nextstrain clade and Pango lineage labels at clade roots. As of June 9, 2021, our SARS-CoV-2 MAT consists of 834,521 sequences and provides a comprehensive view of the virus’ evolutionary history using public data. We also present matUtils – a command-line utility for rapidly querying, interpreting and manipulating the MATs. Our daily-updated SARS-CoV-2 MAT database and matUtils software are available at http://hgdownload.soe.ucsc.edu/goldenPath/wuhCor1/UShER_SARS-CoV-2/ and https://github.com/yatisht/usher, respectively.

## Introduction

The COVID-19 pandemic has witnessed unprecedented levels of genome sequencing for a single pathogen (Hodcroft et al. 2021). Since the onset of the pandemic in late 2019, over a million SARS-CoV-2 genomes have been sequenced worldwide, and tens of thousands of new genomes are being shared on various data repositories every day (Maxmen 2021). This data has enabled scientists to closely track the evolution of the virus and study its transmission dynamics at global and local scales (Deng et al. 2020; Chaillon and Smith 2021; da Silva Filipe et al. 2021). However, the scale of this data is posing serious computational challenges for comprehensive phylogenetic analyses (Hodcroft et al. 2021). Platforms like Nextstrain (Hadfield et al. 2018) have been invaluable in studying viral transmission networks and genomic surveillance efforts, but they only provide subsampled SARS-CoV-2 trees consisting of a tiny fraction of available data, omitting phylogenetic relationships with most available sequences. A single, comprehensive SARS-CoV-2 reference tree of all available data could not only facilitate detailed and unambiguous phylogenetic analyses at global, country and local levels, but may also help promote consistency of results across different research groups (Turakhia et al. 2020).

Besides the computational challenges, the massive volume of SARS-CoV-2 data is also posing numerous data sharing challenges with existing file formats, such as Fasta or Variant Call Format (VCF), which are bulky and necessitate network speeds and computational capabilities that are beyond the reach of many research and scientific groups involved in studying SARS-CoV-2 evolution and transmission dynamics worldwide.

## New Approaches

In this work, we simultaneously address the issue of maintaining a comprehensive SARS-CoV-2 reference tree and its associated data processing, data sharing and computational analysis challenges. Specifically, we are maintaining and openly sharing a daily-updated database of mutation-annotated trees (MATs) containing global SARS-CoV-2 sequences from public databases, including annotations for Nextstrain clades (Hadfield et al. 2018) and Pango lineages (Rambaut et al. 2020) (Supplementary Figure 1). The MAT is an extremely efficient data format proposed recently (Turakhia et al. 2021) which can facilitate the sharing of extremely large genome sequence datasets – an uncompressed MAT of 834,521 SARS-CoV-2 public sequences requires only 65 MB to store, and encodes more information than is contained in a 43 GB VCF and a 38 MB Newick file combined.

To accompany this database, we present matUtils – a toolkit for querying, interpreting and manipulating the MATs. Using matUtils, common operations in genomic surveillance and contact tracing efforts, including annotating a MAT with new clades, extracting subtrees of the most closely-related samples, or converting the MAT to standard Newick or VCF format can be performed in a matter of seconds to minutes even on a laptop. We also provide a web interface for matUtils through the UCSC SARS-CoV-2 Genome Browser (Fernandes et al. 2020). Together, our SARS-CoV-2 database and matUtils toolkit can simultaneously democratize and accelerate pandemic-related research.

## Results and Discussion

### A daily-updated mutation-annotated tree database of global SARS-CoV-2 sequences

To aid the scientific community studying the mutational and transmission dynamics of the SARS-CoV-2 virus and its different variants, we are maintaining a daily-updated database of SARS-CoV-2 mutation-annotated trees (MATs) composed of public data. Starting with the final Newick tree release dated November 13, 2020, of Rob Lanfear’s sarscov2phylo (https://github.com/roblanf/sarscov2phylo) that is re-rooted to Wuhan/Hu-1 (GenBank MN908947.3, RefSeq NC_045512.2), we have set up an automated pipeline to aggregate public sequences available through GenBank (Clark et al. 2007), COG-UK (Nicholls et al. 2020), and the China National Center for Bioinformation on a daily basis and incorporate them into our MAT using UShER (see Supplementary Methods). GISAID data (Shu and McCauley 2017) is not included in our MATs because its usage terms do not allow redistribution. We also use the *matUtils annotate* command (see Supplementary Methods) to add Nextstrain clade and Pango lineage annotations to individual branches of our MAT. As of June 9, 2021, our MAT consists of 834,521 sequences, includes 14 Nextstrain clade and 895 Pango lineage annotations for all samples, and is only 65 MB, or 14 MB in its gzip-compressed form (Supplementary Figure 1, Supplementary Table S1). To our knowledge, this is the most comprehensive representation of the SARS-CoV-2 evolutionary history using publicly available sequences. It can be freely used to study evolutionary and transmission dynamics of the virus at global, country and local levels.

### matUtils provides a wide range of functions to analyze and manipulate mutation-annotated trees

**Figure 1:**
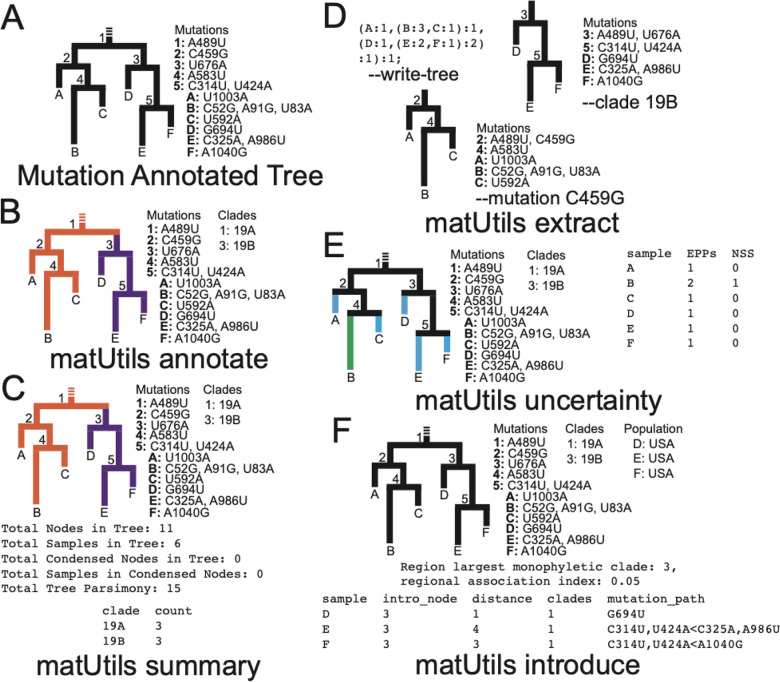
matUtils functions enable fast, user-friendly analysis of mutation-annotated trees (MATs). **(A)**: An example MAT with tree topology corresponding to the MAT on the left and the mutation annotations on each node shown on the right. **(B)**: *matUtils annotate* allows the user to annotate internal nodes with clade names. In this example, nodes 1 and 3 are annotated with clade names 19A and 19B, respectively. **(C)**: *matUtils summary* outputs sample-, clade-, and tree-level statistics for the input MAT. **(D)**: *matUtils extract* allows users to convert a MAT (of panel C in this example) to Newick format (left), subset the MAT for a specified clade (right) or mutation (bottom), among other functions. **(E)**: *matUtils uncertainty* outputs parsimony scores, equally parsimonious placements (EPPs) and neighborhood size scores (NSS) for each sample. **(F)**: *matUtils introduce* takes as input a list of samples of interest, and outputs their predicted introduction nodes and paths, along with confidence scores for both the introduction and parent node (not shown). Using the -*a* flag, the user can also determine the largest monophyletic clade and regional association index associated with the input population. Where relevant for all functions shown, text outputs are displayed in fixed-width fonts for differentiation from attributes internal to the input protobuf.

We have created a high-performance command line utility called matUtils for performing a wide range of operations on MATs for rapid interpretation and analysis in genomic surveillance and contact tracing efforts. matUtils is distributed with the UShER package (Turakhia et al. 2021) and uses the same mutation-annotated tree (MAT) format as UShER. matUtils is organized into five different subcommands: annotate, summary, extract, uncertainty and introduce (Figure 1), described briefly below. We provide detailed instructions for the usage of each module on our wiki (https://usher-wiki.readthedocs.io/en/latest/matUtils.html).

#### Annotate:

This function provides the ability to annotate the clades in the tree. One of the central uses of phylogenetics during the pandemic is to trace the emergence and spread of new viral lineages. Nextstrain (Hadfield et al. 2018), Pango (Rambaut et al. 2020) and GISAID (Shu and McCauley 2017) provide different nomenclatures for SARS-CoV-2 variants that have been used widely in genomic surveillance. Our MAT format provides the ability to annotate internal branches of the tree with an array of clade names, one for each clade nomenclature. Clades can be annotated on a MAT using *matUtils annotate* in two ways: (i) directly providing the mappings of each clade name to its corresponding node or (ii) providing a set of representative sample names for each clade from which the clade roots can be automatically inferred (see Supplementary Methods). Both ways of annotating ensure that the clades remain monophyletic, but we use the second approach to label Nextstrain clades and Pango lineages in our SARS-CoV-2 MAT database since it can be automated using available data (see Supplementary Methods). *matUtils annotate* has high congruence with Nextstrain clades and Pango lineage annotations (Supplementary Table S1).

Once clades are annotated on a MAT, the UShER placement tool (Turakhia et al. 2021) can assign each newly placed sequence to its corresponding Pango lineage, and this being used as a feature in Pangolin 3.0 (https://github.com/cov-lineages/pangolin/releases/tag/v3.0) to perform clade assignments in a fully phylogenetic framework.

#### Summary:

This function provides a brief summary of the available data in the input MAT file and is meant to serve as a typical first step in any MAT-based analysis. It provides a count of the total number of samples in the MAT, the number of annotated clades, the size of each clade, the total parsimony score (i.e. the sum of mutation events on all branches of the MAT), the number of distinct mutations, clade assignments for each sample, and other similar statistics.

#### Extract:

**Figure 2:**
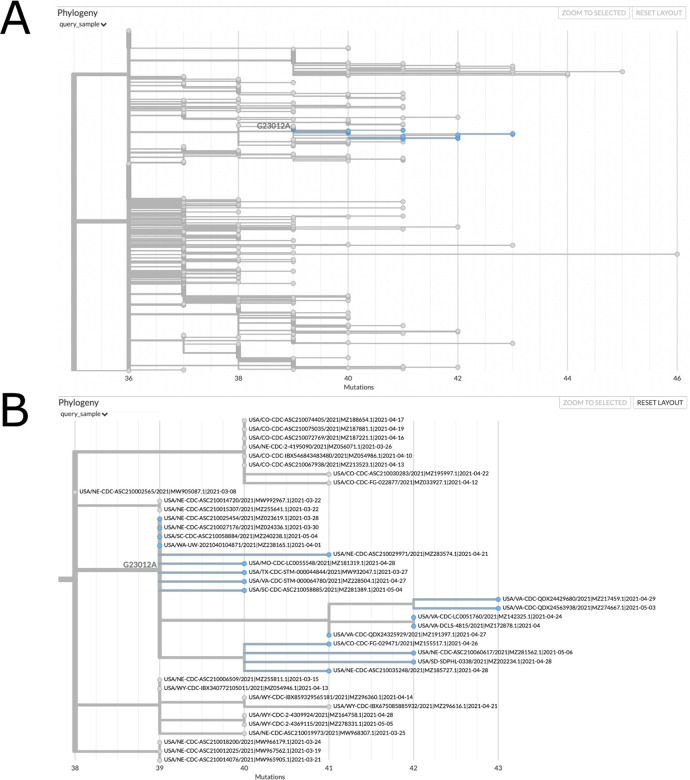
matUtils can generate informative visuals with Auspice. The above trees represent a clade of related B.1.1.7 samples from the USA which secondarily acquired the potentially important spike protein mutation E484K, which is caused by the nucleotide mutation G23012A. These trees were obtained by running the command “matUtils extract -i public-2021-06-09.all.masked.nextclade.pangolin.pb.gz -c B.1.1.7 -m G23012A -H “(USA.*)” -N 500 -j clade_trees -d clade_out”, which selects all samples from clade B.1.1.7 which acquired this mutation and are from the USA, then identifies the minimum set of five hundred sample subtrees which contain all of these samples, creating an Auspice v2 format JSON for each subtree (Hadfield et al 2018). This results in thirty-five distinct subtree JSON files of five hundred samples each in the output directory. Panel A represents the entirety of subtree six as viewed with Auspice (Hadfield et al 2018), including blue highlights and a branch label where our mutation of interest occurred. Panel B is zoomed in on this subtree and its sister clade; at this scale we can read individual sample names and observe that this specific strain has been actively spreading in the United States during April 2021.

Many SARS-CoV-2 phylodynamic studies involve restricting the analysis to a smaller tree of interest, such as a tree of sequences belonging to a particular geographic region or clade. It can be computationally challenging to identify samples most closely related to a given sample or cluster from over a million other sequences, or infer individual subtrees, but it is straightforward to retrieve subtrees from a comprehensive phylogeny. *matUtils extract* provides an efficient and robust suite of options for subtree selection from a MAT that could transform viral genomic surveillance efforts. A user can use *matUtils extract* to subsample a MAT to find samples that contain a mutation of interest, are members of a specific clade, have a name matching a specific regular expression pattern (such as the expression “(IND*|India*)” to select samples from India), among other criteria (see Supplementary Methods). *matUtils extract* also includes options for pruning low-quality sequences from a MAT, such as those with an unusually high parsimony score. Notably, *matUtils extract* can produce an output Auspice v2 JSON that is compatible with the Auspice tree visualization tool (Hadfield et al. 2018) (Figure 2, Supplementary Methods). *matUtils extract* can also convert a MAT into other file formats, such as a Newick for its corresponding phylogenetic tree and a VCF for its corresponding genome variation data.

#### Uncertainty:

A fundamental concern in SARS-CoV-2 phylogenetics is topological uncertainty (Hodcroft et al. 2021). This is especially true for public health, where sample level uncertainty statistics convey the reliability of genomic contract tracing. matUtils provides such a statistic through its uncertainty function, which computes the number of equally parsimonious placements (Turakhia et al. 2020) that exist for each specified sample in the input MAT. Importantly, matUtils also allows the user to calculate equally parsimonious positions for already placed samples. This is accomplished by pruning the sample from the tree and placing the sample back to the tree using the placement module of UShER (Turakhia et al. 2021) (see Supplementary Methods). *matUtils uncertainty* additionally records the number of mutations separating the two most distant equally parsimonious placements, reflecting the distribution of placements across the tree (see Supplementary Methods). The output file is compatible as “drag-and-drop” metadata with the Auspice platform which allows for a rapid visualization of sample placement uncertainty (Supplementary Figure 2)

#### Introduce:

Public health officials are often concerned about the number of new introductions of the virus genome in a given country or local area. To aid this analysis, *matUtils introduce* can calculate the association index (Wang et al. 2001) or the maximum monophyletic clade size statistic (Salemi et al. 2005; Parker et al. 2008) for arbitrary sets of samples, along with simple heuristics for approximating points of introduction into a region (see Supplementary Methods).

### matUtils enables rapid analysis of a comprehensive SARS-CoV-2 global tree and its web interface

The sheer scale of genomic data collected during the ongoing SARS-CoV-2 pandemic has necessitated the development of new tools for effective phylogenetic analysis. The matUtils toolkit is designed to scale efficiently to SARS-CoV-2 phylogenies containing millions of samples. Using matUtils, common pandemic-relevant operations described in the earlier section can be performed in the order of seconds to minutes with the current scale of SARS-CoV-2 data (Supplementary Tables S2–S9). For example, it takes only 5 seconds to summarize the information contained in our 06/09/2021 SARS-CoV-2 MAT of 834,521 samples and only 15 seconds to extract the mutation paths from the root to every sample in the MAT (Supplementary Table S2). Since matUtils is primarily designed to work with the newly-proposed and information-rich MAT format, it does not have direct counterparts in other bioinformatic software packages currently, but its efficiency is similar or better than state-of-the-art tools that offer comparable functionality (Supplementary Tables S2–S9). For example, matUtils is able to resolve polytomies in a 834,521 sample tree in 9 seconds, a task which takes over 37 minutes using ape (Paradis and Schliep 2019) (Supplementary Table S3). matUtils is also very memory-efficient, requiring less than 1.4 GB of main memory for most tasks, making it possible to run even on laptop devices.

Certain functions of matUtils (such as extracting subtrees of provided sample names or identifiers) have also been ported to UCSC SARS-CoV-2 Genome Browser (Fernandes et al. 2020) and are available from https://genome.ucsc.edu/cgi-bin/hgPhyloPlace. This provides a user-friendly web interface to public health officials and researchers working on combating the pandemic.

Our database and utility fill a critical need for open, public, rapid analysis of the global SARS-CoV-2 phylogeny by health departments and research groups across the world, with highly-efficient file formats that do not require high speed internet connectivity or large storage devices, and tools capable of rapidly performing large-scale analyses on laptops.

## Supplementary Material

Supplement 1

1
