# Supplementary material for: A daily-updated database and tools for comprehensive SARS-CoV-2 mutation-annotated trees
Source: bioRxiv. 2021 Jul 13:2021.04.03.438321. Originally published 2021 Apr 4. Preprint. [Version 2] doi: 10.1101/2021.04.03.438321 (PMC8020970; doi:10.1101/2021.04.03.438321)
Supplement: 1 [file NIHPP2021.04.03.438321V2-supplement-1.pdf]

bioRxiv preprint doi: https://doi.org/10.1101/2021.04.03.438321; this version posted July 13, 2021. The copyright holder for this preprint (which was not certified by peer review) is the author/funder, who has granted bioRxiv a license to display the preprint in perpetuity. It is made available under aCC-BY 4.0 International license.

Supplementary Methods

Maintaining a daily-updated mutation-annotated tree database of global SARS-CoV-2 sequences

We are maintaining a daily-updated mutation-annotated tree (MAT) database of global SARS-CoV-2 sequences at http://hgdownload.soe.ucsc.edu/goldenPath/wuhCor1/UShER_SARS-CoV-2/. Our database is organized into sub-directories sorted by year, month and date. To update the MATs daily, we have set up a CRON job on a server at UCSC which downloads SARS-CoV-2 sequences daily from GenBank (Clark et al. 2007) and COG-UK (Nicholls et al. 2020) (see https://github.com/ucscGenomeBrowser/kent/blob/master/src/hg/utils/otto/sarscov2phylo/updatePublic.sh which calls other scripts in the same directory). We also include 253 sequences downloaded from the China National Center for Bioinformation (https://bigd.big.ac.cn/ncov/release_genome) in October 2020 that are not associated with GenBank IDs.

New sequences are added to the previous day's MAT using the UShER placement tool (Turakhia et al. 2021) with options to place the samples in the order of the fewest ambiguous bases and exclude sequences with 5 or more equally parsimonious placements. Previously excluded sequences are reconsidered for placement during each build. We also use *matUtils extract* to prune samples with 30 or more private mutations and those internal branches longer than 30 mutations, as these are highly indicative of error-containing sequences (Mai and Mirarab 2018). The trees are rooted to Wuhan/Hu-1 (GenBank MN908947.3, RefSeq NC_045512.2), and nodes with no associated mutations are collapsed (Turakhia et al. 2021). Our first MAT was created by starting with the last Newick tree release (dated November 13, 2020) of Rob Lanfear's sarscov2phylo (Lanfear and Mansfield 2020) containing 82,358 public sequences, adding the later additional public sequences using UShER. Each MAT is then annotated with Nextstrain clade and Pango lineage annotations using *matUtils annotate -c* with a file containing representative sequences for each clade/lineage. For Nextstrain clades, Nextclade assignments (https://github.com/nextstrain/nextclade) for all sequences are used. For Pango lineages, designated lineage representative sequences from https://github.com/cov-lineages/pango-designation/ are mapped to the corresponding public sequence IDs where possible.

In addition to MATs, we provide in each sub-directory: (i) a Variant Call Format (VCF) file containing the genotypes of public sequences, generated from the corresponding MAT with matUtils extract such that missing or ambiguous bases have been imputed by UShER using maximum parsimony (Turakhia et al. 2021), (ii) a Newick file also generated from the corresponding MAT using *matUtils extract* (iii) a tab-separated file containing information about each public sequence e.g. collection date, location, Nextstrain clade and Pango lineage, (iv) a tab-separated file with Nextstrain clades assigned to sequences by Nextclade

bioRxiv preprint doi: https://doi.org/10.1101/2021.04.03.438321; this version posted July 13, 2021. The copyright holder for this preprint (which was not certified by peer review) is the author/funder, who has granted bioRxiv a license to display the preprint in perpetuity. It is made available under aCC-BY 4.0 International license.

(https://github.com/nextstrain/nextclade) and (v) a tab-separated file with Pango lineages assigned to sequences by pangolin (https://github.com/cov-lineages/pangolin).

Our script to update the MAT daily is available at https://github.com/ucscGenomeBrowser/kent/blob/master/src/hg/utils/otto/sarscov2phylo/updateCombinedTree.sh.

matUtils: Design Overview

matUtils is implemented using the C++ programming language and is developed and maintained within the phylogenetic placement package of UShER (Turakhia et al. 2021), since matUtils shares the core mutation-annotated tree (MAT) data structure with UShER, which helps us ensure cross-compatibility of both tools. matUtils complements UShER through its ability to analyze and manipulate the MAT output but can be used as a standalone phylogenetics tool independent of UShER. matUtils and UShER can be installed together via (i) a Docker container (https://hub.docker.com/repository/docker/yatisht/usher), (ii) the Conda package manager using the bioconda (Grüning et al. 2018) channel (http://bioconda.github.io/recipes/usher/README.html) or (iii) the installation scripts that we provide on our GitHub repository (https://github.com/yatisht/usher) for some recent Linux and MacOS releases. Detailed installation and usage instructions are available on our wiki: https://usher-wiki.readthedocs.io/en/latest/matUtils.html. Several matUtils functions have multi-threaded parallel implementations through Intel's Thread Building Blocks library (https://github.com/oneapi-src/oneTBB).

matUtils: Implementation details

Annotate: *matUtils annotate* is designed to annotate clades on the internal branches of the MAT. Our MAT format (specified in https://github.com/yatisht/usher/blob/master/parsimony.proto) provides an ability to annotate internal branches with an array of clade names, one for each clade nomenclature. Each run of *matUtils annotate* extends the clade name array size in a MAT by one to accommodate a new nomenclature. Only the node corresponding to a clade root is labeled with its clade name, as descendants of that node can be automatically inferred to belong to that clade. Clades can be nested, so that each sequence can be assigned to a clade corresponding to the lowest-level clade root to which it is a descendant. *matUtils annotate* provides two different ways to annotate clades in a MAT. Both ways, by design, ensure that all clades remain monophyletic. In the first, a user can directly provide the internal node identifiers corresponding to the root of each clade. In the second, a user can provide a list of representative sequences for each clade, such as training data for Pango lineages (https://github.com/cov-lineages/pango-designation), from which the clade root can be inferred in the tree. Not all sequences in the tree need to be designated by a clade. Since the training data is imperfect, and the representative sequences for lineages are sometimes non-monophyletic in our tree, we have found the simple approach of using the most recent common ancestor (MRCA) does not yield accurate results. The *matUtils annotate* inference method works instead by first building a "consensus" sequence (where, by

bioRxiv preprint doi: https://doi.org/10.1101/2021.04.03.438321; this version posted July 13, 2021. The copyright holder for this preprint (which was not certified by peer review) is the author/funder, who has granted bioRxiv a license to display the preprint in perpetuity. It is made available under aCC-BY 4.0 International license.

default, the consensus sequence requires an allele to be present in at least 80% of representative sequences, with lower frequency alleles marked as ambiguous) for each clade and finding its phylogenetic placement using UShER's placement module to obtain the clade root. When multiple equally parsimonious placements are available for the clade root, the algorithm uses a heuristic formula to compute the "best fit" for the training data, which rewards the placement containing a higher proportion of samples designated by that clade in the training data and penalizes descendants designated by some other clade in the training data. When the same root is found for multiple clades, the clade with fewest equally parsimonious placements, followed by the number of representative sequences in the training data, is prioritized.

Extract: The *extract* subcommand acts as a simple prebuilt pipeline with three distinct stages. The first of these, sample selection, collects the set of samples which fulfill each of the conditions indicated by input parameters, then gets the intersection of these sets to identify samples which fulfill all conditions specified on the command. Multiple conditions can be simultaneously specified in a single command for selecting samples, such as clade membership, maximum parsimony score, presence of a particular mutation, and whether the sample name matches a specific regular expression pattern, among others. The second stage edits the input tree object to generate the indicated subtree, either by pruning excluded samples or by generating a subtree in a parallelized fashion, depending on the size of the chosen sample input. The third stage generates each of the requested output files representing the final tree. These files include Newick for pure tree information, parsimony-resolved VCF for variation information, and Auspice v2 format JSON for both (Hadfield et al. 2018). VCF production is parallelized for efficiency with large sample selections. A sample metadata table in CSV or TSV format can be incorporated into the JSON output. The full list of options can be found at our wiki: https://usher-wiki.readthedocs.io/en/latest/matUtils.html.

Uncertainty: *matUtils uncertainty* can calculate two different metrics for characterizing the phylogenetic certainty of a sample placement. The first metric is "equally parsimonious placements" (EPPs), which is the number of places on the tree a sample could be placed without affecting the parsimony score. An EPP score of 1 indicates a high placement certainty of a sample in its local neighborhood in that there is a single most parsimonious placement location for that sample on the entire tree, and a higher EPP score suggests the sample placement is less certain. This metric is calculated by computing the number of most parsimonious placements after remapping the input sample(s) against the same tree (disallowing it from mapping to itself) with UShER's optimized placement module. About 85% of samples in our SARS-COV-2 MAT database have an EPP score of 1. The second metric is "neighborhood size score" (NSS), which is the longest distance (in number of edges) between any two equally parsimonious placement locations for a given sample. This metric is complementary to EPPs – when multiple EPPs are possible for a sample, NSS indicates whether the placement uncertainty is restricted to a small neighborhood (small NSS value) or spans a large portion of the tree (large NSS value).

bioRxiv preprint doi: https://doi.org/10.1101/2021.04.03.438321; this version posted July 13, 2021. The copyright holder for this preprint (which was not certified by peer review) is the author/funder, who has granted bioRxiv a license to display the preprint in perpetuity. It is made available under aCC-BY 4.0 International license.

Introduce: *matUtils introduce* is aimed to help epidemiologists and public health officials estimate the number of new introductions of the virus in a given area or country. It includes a command which calculates maximum monophyletic clade size and association index statistics for phylogeographic trait association for user-provided input regions. Maximum monophyletic clade size (Parker et al. 2008) is the largest monophyletic clade of samples which are in the region – it is larger for regions which have relatively fewer introductions per sample and correlates with overall sample size. Association index (Wang et al. 2001) is a more complex metric which performs a weighted summation across the tree accounting for the number of child nodes and the frequency of the most common trait, such as membership in a particular geographical region of interest. Association index is smaller for stronger phylogeographic association and increases with the relative number of introductions into a region. For association index, *matUtils introduce* also performs a series of permutations to establish an expected range of values for the random distribution of samples across the tree. We are also working with epidemiologists currently to expand *matUtils introduce* with new heuristics (currently in an experimental stage but described in more detail on our wiki at: https://usher-wiki.readthedocs.io/en/latest/matUtils.html) for estimating the number of new introductions of the virus in a given area, details of which will appear in a later publication.

Performance benchmarking of matUtils and other phylogenetics software packages

All performance benchmarking experiments were carried out on a Google Cloud Platform (GCP) instance n2d-standard-16 with 16 vCPUs (Intel Xeon CPU E7-8870 v.4, 2.10 GHz) with 64 GB of memory using our public SARS-CoV-2 MAT dated June 9, 2021. matUtils does not have direct counterparts for its ability to work with the mutation-annotated tree (MAT) format, but we compared the performance of matUtils with state-of-the-art tools that offer some comparable functionality on Newick or VCF formats. Specifically, we compared the most recent version of matUtils (version 0.3.1) to newick_utils version 1.6 (Junier and Zdobnov 2010), tree_doctor (from version 1.5 of the phast package; (Hubisz et al. 2011), ape version 5.5 (Paradis and Schliep 2019), and bcftools version 1.7 (Danecek et al. 2011). The exact commands used for each comparison can be found in Supplementary Tables S2-S9, and the input data used for each comparison can be found at https://github.com/bpt26/matutils_benchmarking/.

bioRxiv preprint doi: https://doi.org/10.1101/2021.04.03.438321; this version posted July 13, 2021. The copyright holder for this preprint (which was not certified by peer review) is the author/funder, who has granted bioRxiv a license to display the preprint in perpetuity. It is made available under aCC-BY 4.0 International license.

Supplementary Figures and Tables

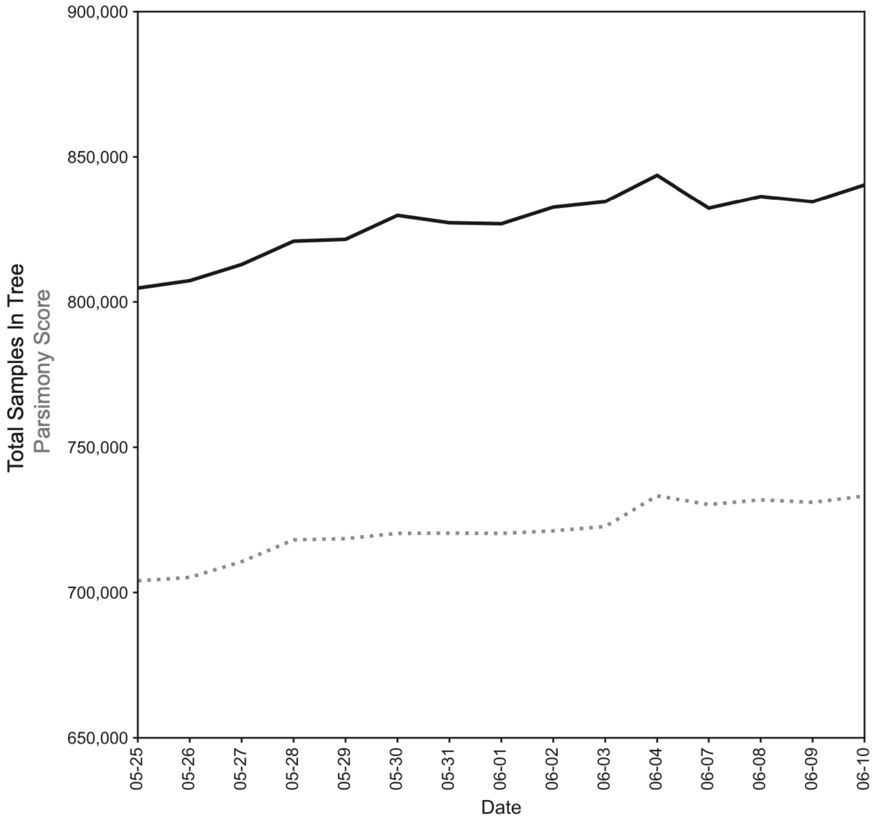

Supplementary Figure 1: Our global phylogeny contains 840,343 samples as of June 10. Our database, containing all high-quality publically available SARS-CoV-2 whole-genome sequences and their clade assignments, is updated daily. As of June 10, our phylogeny contains 840,343 sequences with a total parsimony score of 733,211. Sequences that have 5 or more equally parsimonious placements on the tree are removed at each build (see Supplementary Methods), so the total samples sometimes drop during successive builds.

bioRxiv preprint doi: https://doi.org/10.1101/2021.04.03.438321; this version posted July 13, 2021. The copyright holder for this preprint (which was not certified by peer review) is the author/funder, who has granted bioRxiv a license to display the preprint in perpetuity. It is made available under aCC-BY 4.0 International license.

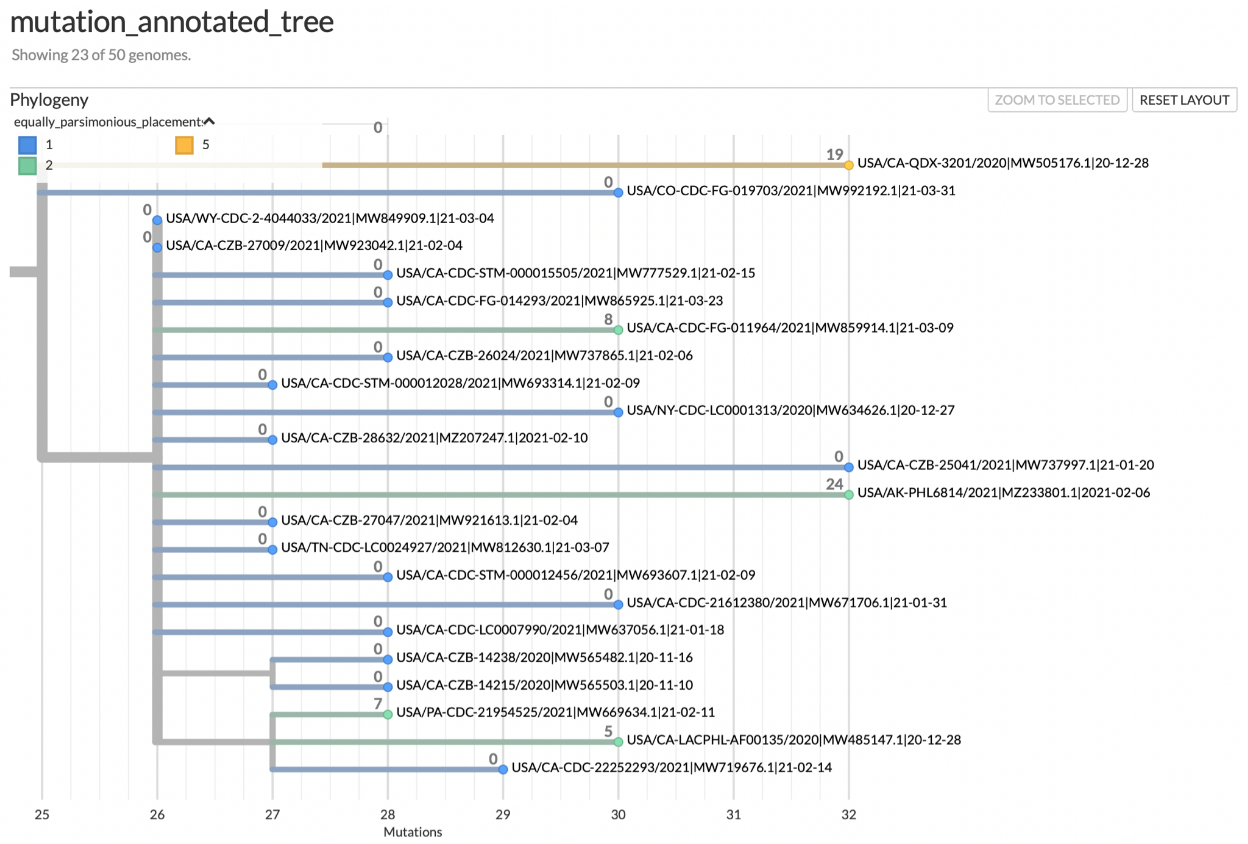

Supplementary Figure 2: matUtils uncertainty statistics reveal low-quality sample placements. This Auspice view of an example subtree is annotated with both equally parsimonious placements (in color) and neighborhood size (branch label integers). 18 of our 23 samples in the subtree have a single placement and a neighborhood size of 0, indicating high placement certainty for those samples. Of the five samples with multiple equally parsimonious placements, one sample has 5 equally parsimonious placements with an NSS value of 19, indicating a high level of placement uncertainty for this sample spanning a relatively large neighborhood.

Group	Number of clades/lineages	Training Size	Test Size	Mean Training Accuracy	Minimum Training Accuracy	Mean Test Accuracy	Minimum Test Accuracy
Nextstrain	14	651446	184877	0.973	0.972	0.971	0.970
Pangolin	895	651446	184877	0.881	0.881	0.881	0.880

Supplementary Table S1: matUtils annotate can quickly and effectively assign clade lineage roots. This table was generated by taking training data associated with Nextstrain clades and Pango lineages from our public repository (lineageToPublicName.gz and cladeToPublicName.gz), splitting the data 80/20 into training and test sets, and assigning roots based on the 80% selected training data with matUtils annotate on the 06-09-2021 public MAT

bioRxiv preprint doi: https://doi.org/10.1101/2021.04.03.438321; this version posted July 13, 2021. The copyright holder for this preprint (which was not certified by peer review) is the author/funder, who has granted bioRxiv a license to display the preprint in perpetuity. It is made available under aCC-BY 4.0 International license.

tree. Accuracy was scored as the percentage of the training or test set which matches Nextclade or Pangolin assignments. This process was repeated 9 times and mean and minimum accuracy values were collected.
